# Regional Comprehensive Economic Partnership Can Boost Value-Added Trade in Food and Non-Food Sectors in Asia–Pacific Economies

**DOI:** 10.3390/foods13132067

**Published:** 2024-06-28

**Authors:** Wei Wei, Tariq Ali, Mengge Liu, Guolei Yang

**Affiliations:** 1 School of Economics and Management, Qingdao Agricultural University, Qingdao 266109, China; weiwei2023@qau.edu.cn; 2 School of Economics and Management, Jiangxi Agricultural University, Nanchang 330045, China; 3 School of Economics and Management, China University of Petroleum, Qingdao 266580, China; 4 Academy of National Food and Strategic Reserves Administration, Beijing 100037, China

**Keywords:** RCEP, value added trade, CGE model, Asia–Pacific economies, food products

## Abstract

This study examines the effects of the Regional Comprehensive Economic Partnership (RCEP) on the value-added trade of food and non-food sectors. This study uses a global computable general equilibrium (CGE) model coupled with an extension module for the origin decomposition of value-added flows embodied in gross trade. The results suggest that by cutting down tariff and non-tariff barriers, the RCEP would significantly stimulate the economies of and gross trade among Asia–Pacific countries involved in the agreement. The potential benefits of the RCEP will be overestimated if we ignore the origin of value added and measure the benefits by gross exports. The domestic components of bilateral value-added flows between RCEP members would increase greatly, indicating an increasingly integrated value chain between RCEP members. Import taxes and non-tariff barriers for processed food, textiles and clothes, and heavy manufacturing are relatively significant in the region, so the RCEP would significantly improve their value-added exports. The domestic component of value-added exports in agricultural products and processed food from RCEP members would be increased significantly, indicating that the closely integrated food value chain boosts the food economies of RCEP members.

## 1. Introduction

The Regional Comprehensive Economic Partnership (RCEP) is expected to establish the largest regional free-trade region and effectively promote trade in Asia–Pacific economies. The ASEAN-led initiative of the RCEP was first launched in November 2012, aimed at achieving a modern, comprehensive, high-quality, and mutually beneficial economic partnership [1,2] by eliminating tariff and non-tariff trade barriers in Asia–Pacific economies. Although India did not join the RCEP in 2019, negotiations to reach an agreement continue among the 15 Asia–Pacific economies, including 10 ASEAN countries (Association of Southeast Asian Nations), China, Japan, South Korea, Australia, and New Zealand. On 15 November 2020, the RCEP agreement was signed by the 15 members, requiring that tariffs be eliminated in the coming ten years and that the openness of commodity trade will exceed 90% [1].

The RCEP will establish a giant trading block, covering a population of 2.25 billion and with a purchasing power parity (PPP) gross domestic product (GDP) of USD 38.33 trillion. It accounted for about 31.72% of the world’s GDP in 2018 [3]. The possible economic benefits from the RCEP are expected to improve the economies of the member countries and act as an effective measure to mitigate the economic damages caused by the United States–China trade war and the COVID-19 pandemic [4]. However, the potential success of the RCEP will depend on the extent to which the member countries can reach a consensus [5,6], which is predominantly dependent upon weighing each country’s benefit against its loss. Therefore, a comprehensive assessment of the effects of the RCEP in Asia–Pacific economies is necessary.

Previous studies assessing the effects of the RCEP focused mostly on the changes in the national GDP and bilateral commodity trade of member countries, finding that almost all RCEP members would benefit from the agreement, while nearly all non-participants would experience losses [7,8,9,10,11]. Some studies also found that the RCEP will likely lead to structural change for almost all of its members in manufacturing and services and detract from the agriculture and food sectors [12,13]. Kawasaki [8] found that the developing economies involved in the RCEP, such as ASEAN countries, would enjoy relatively larger GDP gains than developed economies. On the other hand, Petri et al. [14] noted that the RCEP would generate global GDP gains of USD 286 billion. Moreover, Lee and Itakura [13], Kikuchi et al. [15], and Petri et al. [14] also incorporated reductions in non-tariff barriers (NTBs), using equivalent ad valorem tariffs of imports, and found larger benefits from the RCEP than from tariff reduction only. However, most of the previous studies did not explicitly examine the effects of the RCEP on food and non-food trade in Asia–Pacific economies.

Analyzing the value-added flows embodied in bilateral trade under the RCEP can reveal new insights not covered in previous studies. The recent literature on this topic has greatly enriched our knowledge of underlying value-added flows in gross trade values [16,17,18,19], the position and participation of a country or sector within the global value chain [18,20,21], and the links through which foreign demand activates domestic production [19,22]. Studies on the global value chain measure the value-added flows embodied in bilateral trade at the macro level using different approaches. According to Johnson [23], value-added trade can be assessed by decomposing the value-added flows embodied in final goods or gross exports. Although some studies have combined multi-regional input–output (MRIO) tables with value-added decomposition in the context of trade policy [24,25,26,27], integrated assessment of the effect of the RCEP from the perspective of value-added trade is rather scarce. Itakura and Lee [28] modified the import demand module of the GTAP model and analyzed the impacts of the CPTPP and the RCEP from the global value chain perspective, considering reductions in both tariff and non-tariff barriers. However, they did not split the value-added trade of food and non-food products from gross trade and decompose value-added trade into domestic and foreign components.

To bridge the research gaps, this study employs a computable general equilibrium (CGE) modeling framework, specifically the Global Trade Analysis Project (GTAP) model, along with an extension module to decompose the value-added trade flows of food and non-food products. We quantitatively examine the effects of the RCEP on food and non-food products in Asia–Pacific economies from the perspective of value-added trade. Additionally, the effects of India’s potential participation in the future are examined. The benefits gained by Asia–Pacific countries in the global value chain are revealed by decomposing the components of value-added trade. The study adds to the existing literature in two areas. First, by decomposing the value-added flows by origin, this study estimates the domestically originated value added that is regarded as the actual benefit gained by each RCEP member. In general, the domestically originated components of value-added exports are much lower than those of gross exports, which may indicate that the effects of the RCEP are unintentionally overestimated, at least partly based on gross trade values. Second, this paper examines the effects of the RCEP on food and non-food products, adding a novel viewpoint to the literature. Previous studies mostly evaluated the effects of the RCEP on macroeconomic factors (such as GDP) and gross trade, despite the great significance of the food economy for Asia–Pacific countries.

The remainder of this paper is organized as follows. Section 2 introduces the changing trends and characteristics of international trade among RCEP countries. Section 3 describes the methodology. Section 4 discusses the results, and the last section concludes this study.

## 2. International Trade among RCEP Members and India

**Figure 1:**
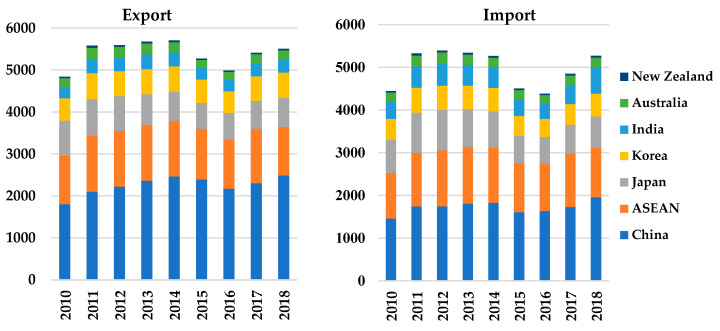
International trade values for RCEP countries and India from 2010 to 2018 (USD billion). Source: UN COMTRADE, 2020 [29].

In the past decade, rapid economic growth and trade liberalization (achieved by reducing tariff and non-tariff barriers) fostered the total imports and exports of Asia–Pacific economies. According to the UN COMTRADE database [29], for example, China’s total exports increased from USD 1804 billion in 2010 to USD 2487 billion in 2018, with an annually averaged growth rate of 4.3%, and its total imports simultaneously rose from USD 1455 billion in 2010 to USD 1958 billion in 2018, with an annually averaged growth rate of 4.2% (Figure 1). The rapidly rising trade figures may reflect that China imported many raw materials and intermediate inputs to produce the exported commodities, owing to the production transfer from developed countries. Although both the export and import values of ASEAN countries and Korea increased from 2010 to 2018, their growth rates were much lower than those of China and New Zealand. At the same time, Japan and Australia experienced rising total imports and decreasing total exports. Japan’s total exports decreased from USD 836 billion in 2010 to USD 693 billion in 2018 as a result of the increasingly intensive competition in the global market and the production redistribution of Japanese multinational enterprises to China, India, and ASEAN countries. In contrast, India’s total exports and imports increased rapidly, with annually averaged growth rates of 4.2% and 7.4%, respectively.

As for the trade structure, except for Australia and New Zealand, the commodity exports of RCEP members and India are dominated by heavy manufacturing. Korea and Japan exported heavy manufacturing products worth USD 669.5 and 576.3 billion, respectively, in 2018, accounting for over 95% of their total commodity exports (Table 1). Heavy manufacturing products also account for over 75% of total commodity exports from China, India, and ASEAN countries. However, their structures of commodity exports are more complicated. In 2018, China exported textiles and clothing worth USD 282.0 billion and light manufacturing products worth USD 174.8 billion, accounting for 7.7% and 7.4% of total commodity exports, respectively. Extraction products and processed food accounted for 7.0% and 8.6% of total commodity exports from ASEAN countries, respectively.

In contrast, heavy manufacturing products accounted for relatively low shares of total commodity exports from Australia (26.5%) and New Zealand (16.7%). The extraction products accounted for 54.7% of total commodity exports for Australia, followed by processed foods (9.4%) and agricultural products (7.6%). However, for New Zealand, agricultural products and processed food jointly accounted for 73.6% of its total commodity exports (Table 1). Australia’s and New Zealand’s exports are dominated by primary products, indicating that they could benefit most from international trade.

Relative to exports, all the RCEP members have a high proportion of imported heavy manufacturing products. China, Korea, and Japan have similar structures for commodity imports, with heavy manufacturing products accounting for around 60% and extraction products accounting for over 20% in 2018. Meanwhile, heavy manufacturing products account for 79.3% of total commodity imports by ASEAN countries. Interestingly, heavy manufacturing products accounted for 77.8% and 70.8% of total commodity imports by Australia and New Zealand, respectively—significantly higher values than their shares of commodity exports. India’s structure of commodity imports is quite similar to that of China, Japan, and Korea; heavy manufacturing and extraction products accounted for 57.5% and 34.9% of India’s total imports.

Food trade also plays an important role in integrating Asia–Pacific economies. Agricultural products and processed food are the biggest exports for New Zealand, accounting for 17.3% and 56.33% of its total exports in 2018, as it has abundant land resources. Meanwhile, agricultural products and processed food accounted for 7.6% and 9.4% of Australia’s total exports. Although the exports of agricultural products were relatively small for ASEAN countries and India, processed food accounted for over 8% of their total exports. Meanwhile, the proportions of agricultural products and processed food exports were relatively small for China, Japan, and Korea, as they are highly populated but have relatively fewer endowments for food production. However, agricultural products and processed food accounted for a much higher proportion of total imports for China, Japan, and Korea, accounting for over 7%, 10%, and 6% of their total imports, respectively. These countries have to import many food products to feed their residents and are highly dependent on the global food market. Meanwhile, processed foods are the major imports in Australia and New Zealand. Around 10% of total imports for New Zealand were attributed to processed foods. Therefore, the trade of agricultural products and processed food is significant for the economic integration of Asia–Pacific economies, which has been ignored in previous studies.

## 3. Model and Scenario Setting

### 3.1. The GTAP Model

The Global Trade Analysis Project (GTAP) model is a multi-regional, multi-sectoral general equilibrium model. With a long history of systematic improvements, the GTAP model provides an effective tool for analyzing various trade-related issues, such as the environment, population, energy, and climate change [13,30]. It is a comparative static analysis model, and it assumes that the market is completely competitive and the returns to scale of production remain unchanged. Based on these theoretical assumptions, producers are assumed to maximize profits while consumers are assumed to maximize their utility. Total supply and total demand are in equilibrium, and they jointly determine the values of endogenous variables, such as prices, wages, and capital and land rents. All economies (i.e., countries and regions) connect with each other via commodity trade.

The GTAP model used here was built based on the latest version (V10) of the GTAP database, which was constructed from the IO tables of 141 countries and regions worldwide with 2014 as the base year. The GTAP database contains 65 sectors and 5 primary production factors. For this study, we aggregated original GTAP regions and sectors to facilitate the display and explanation of the simulation results. The 141 countries and regions were aggregated into 12 regions, which specify the RCEP economies and their major trade partners (see the regional mapping in Appendix A). The 65 production sectors were aggregated into 7 sectors, maintaining the major tradable commodities among RCEP members (see the sectoral mapping in Appendix A). The five types of original primary factors were aggregated into four categories (land, capital, labor, and natural resources). All the parameters, such as the substitution elasticity for domestic and imported commodities and the substation elasticity for regional allocation of imports, were obtained from the GTAP V10 database (see Appendix B).

### 3.2. The Extended Module for Value-Added Trade

We adopted the approach Antimiani et al. [31] developed to obtain the value-added multipliers for the decomposition analysis. The GTAP model is based on a complete IO accounting framework that considers all sources and uses of each good. Some manipulations are required to decompose the valued-added trade from the gross trade flows, tracking the movement of intermediate inputs across each border, based on the work by Peters et al. [32] and Antimiani et al. [31]. They converted the GTAP database into a multi-regional IO table. In the standard GTAP model, the sourcing of imports occurs at the border, providing information on the total purchases of intermediate inputs by firms (domestic and imported) and of goods by households and governments, and for investment (domestic and imported), but without attributing the bilateral trade to the agent (e.g., firms or final consumption). To overcome this limitation, Antimiani et al. [31] assumed that all uses of a commodity are sourced in the same way (the proportionality assumption), as suggested by Daudin et al. [33], Johnson and Noguera [16], and Lejour et al. [34].

Let *i* and *j* = 1, …, *N* index sectors, and *s*, *r* =1, …, *C* index countries. As for the standard GTAP notation, we define *VMMD* as the export value of commodity *i* from region *s* to region *r* at market prices. In order to recover the bilateral delivery of intermediates used by sectors, we use the shares of imports used by sectors from the country’s total imports and apply them to bilateral trade.

(1) $$VXIMS_{ij}^{sr}=SHRIFM_{ij}^{r}\times VXMD_{i}^{sr}$$

where *SHRIFM* is the share of import *i* used by sector *j* in region *r* and *VXMD* is the import value of intermediate input *i* from region *r* to region *s*. Hence, *VXIMS* is the value of the intermediate input *i* from region *s* used by sector *j* in region *r*, evaluated at market prices in *s*.

The transport margins, which are not associated with particular commodities and routes in the standard GTAP model, are allocated to the providing countries, assuming that the use of international transport services by each route is proportional to the countries’ contribution to transport service supply. The countries’ shares of exports to the global transport pool (*VTS*) are applied to the international margin for intermediate usage in country r (*VTWRI*).

(2) $$VSTP_{mj}^{sr}=\frac{VST_{m}^{s}}{\sum_{s}VST_{m}^{s}}\times \sum_{i}VTWRI_{mij}^{r}$$

*VSTP* represents the international margin *m* supplied by *s* for *j*’s intermediate usage in region *r*. The distribution of the use of transport services over suppliers is then added to the corresponding margin-producing sector in the supply region (*VTMTXI*).

(3) $$VTMTXI_{ij}^{rs}=\left\{\begin{array}{c} VSTP_{mj}^{sr}\\ 0 \end{array}\right.\begin{array}{c} \;if\\ \;if \end{array}\;\begin{array}{c} i\in MARG\_COMM\\ i\notin MARG\_COMM \end{array}$$

In country *r*, the sectors’ total purchases of intermediate inputs, *Z*, is obtained by adding the domestic component to imports, that is, the value of purchases of domestic commodity *i* used by sector *j* in region *r* (*VDFM*), and the intermediate margin matrix.

(4) $$Z_{ij}^{rs}=\left\{\begin{array}{c} VXIMS_{ij}^{sr}+VDFM_{ij}^{r}+VTMTXI_{ij}^{sr}\\ VXIMS_{ij}^{sr}+VTMTXI_{ij}^{sr} \end{array}\right.\begin{array}{c} if\\ if \end{array}\;\begin{array}{c} r=s\\ r\neq s \end{array}$$

The value of *j*’s output in region *r* (at domestic market prices) is defined as the sum of the total use of intermediate inputs *i* (*Z*) for each producing sector and value added (*VA*). The latter includes the payment to primary factors plus tax revenue, covering taxes on production and output and trade-related taxes incurred by sectors.

(5) $$VOM_{j}^{r}=\sum_{i}\sum_{s}Z_{ij}^{sr}+VA_{j}^{r}$$

The delivery of intermediates used in the production of the receiving country can be expressed as a share of the destination country *r*’s sectoral output.

(6) $$A_{ij}^{sr}=\frac{Z_{ij}^{sr}}{VOM_{j}^{r}}$$

The sectoral value-added shares for country *r* is given by:

(7) $$VSH_{j}^{r}=\frac{VA_{j}^{r}}{VOM_{j}^{r}}$$

In order to obtain the same dimension of the technical coefficient matrix to be used later, we diagonalize the vector of value-added shares and define a diagonal matrix $\hat{VSH}$ with value-added shares in the main diagonal and zero in the off-diagonals:

(8) $${\hat{VSH}}_{ij}^{sr}=\left\{\begin{array}{c} VSH_{j}^{r}\\ 0 \end{array}\right.\begin{array}{c} \;if\\ \;if \end{array}\;\begin{array}{c} r=s,and\;i=j\\ r\neq s,and\;i\neq j \end{array}$$

Next, we define the demand for final consumption in region *r* of commodity *j* from *s* as follows:

(9) $$FIN_{j}^{sr}=\left\{\begin{array}{c} VXCMS_{j}^{sr}+VDPM_{j}^{r}+VDGM_{j}^{r}+\sum_{k,cgds}VDFM_{jk}^{r}+VTMTXC_{j}^{sr}\\ VXCMS_{j}^{sr}+TMTXC_{j}^{sr} \end{array}\right.\begin{array}{c} \;if\\ \;if \end{array}\;\begin{array}{c} r=s\\ r\neq s \end{array}$$

where *VDPM* and *VDGM* are domestic goods *j* demanded in region *r* by private households and government, respectively, and *VDFM* are the domestic goods *j* demanded by sectors *k* in *r*. The term *VTMTXC* represents the margin matrix for final demand. *VXCMS* is the value of imports of commodity *j* from *s* for final consumption in *r* at market prices, obtained by applying the proportionality assumption.

The accounting identity for tradable supplies can be expressed as follows:

(10) $$VOM_{j}^{r}=\sum_{i}\sum_{s}Z_{ji}^{rs}+\sum_{s}FIN_{j}^{rs}VOM_{j}^{r}=\sum_{i}\sum_{s}Z_{ji}^{rs}+\sum_{s}FIN_{j}^{rs}$$

The right-hand side of Equation (10) is equivalent to the row balance condition in the IO analysis. That is, production is completely used up as intermediate or final consumption, either at home or abroad. By rearranging the above equation, the row balance condition can be written as follows:

(11) $$VOM_{j}^{r}=\sum_{i}\sum_{s}A_{ji}^{rs}\times VOM_{i}^{s}+\sum_{s}FIN_{j}^{rs}VOM_{j}^{r}=\sum_{i}\sum_{s}A_{ji}^{rs}\times VOM_{i}^{s}+\sum_{s}FIN_{j}^{rs}$$

The global Leontief inverse (or multiplier) matrix, $L=(I-A{)}^{-1}$, gives the total output requirement, directly and indirectly, worldwide to produce one consumption unit. This represents the ripple effects in an economy where industries are interconnected. The value added associated with each unit of final demand is obtained by post-multiplying the diagonal matrix of value-added shares, $\hat{VSH}$, with the global Leontief inverse, that is, $\hat{VSH}\times L$.

Finally, by indexing the country with *t* = 1, …, *C*, denoting the country of origin of value, the value added that originates in sector *i* of country *t* and is embodied in country *s*’s exports in sector *j* to country *r* is given by $TVA_{ij}^{tsr}$.

(12) $$TVA_{ij}^{tsr}=\hat{VSH_{l}^{t}}\times L_{ij}^{ts}\times VXE_{j}^{sr}$$

where *VXE* represents bilateral exports, excluding intra-regional trade. The domestic value added exported by each sector does not coincide with the gross trade by commodity, for it may be larger or smaller according to the input used by and/or generated from the other sectors of the economy.

By exploiting the information on the origin of value added embodied in trade flows, the export values are decomposed at the sector level into domestic and foreign components of value added generated in their production. The domestic component is sourced both directly from the producing/exporting sector and indirectly from other domestic sectors’ or other countries’ exports. In contrast, the foreign component of value added is generated from other countries producing the imported inputs used in exports. For this, we can decompose bilateral gross exports into three components: the domestic value added (***DVA***), the double-counted term related to the domestic content (***DDC***), and the foreign content (***FVA***). The domestic content of value-added exports measures the contribution a given export makes to an economy’s income. The remainder is the value of imported inputs representing the import content of exports, which contributes to the income of other countries. The DDC could be calculated by introducing the local (or domestic) Leontief inverse, $LOC_{ij}^{ss}=(1-A_{ij}^{ss}{)}^{-1}$, which is computed on the domestic block of the technical coefficient matrix, thus representing intra-country processing only. The difference between the global and local Leontief inverse $\left(L_{ij}^{ss}-LOC_{ij}^{ss}\right)$ gives the portion of the domestic value added that has crossed international borders at least twice.

(13) $$VXE_{j}^{sr}=\sum_{i}\sum_{t}TVA_{ij}^{tsr\;\;\;\;\;\;\;\;\;\;\;\;\;\;\;\;\;\;\;\;\;\;\;\;\;\;\;}\;=\sum_{i}\hat{VSH_{l}^{s}}\times LOC_{ij}^{ss}\times VXE_{j}^{sr}+\sum_{i}\hat{VSH_{l}^{s}}\times (L_{ij}^{ss}-LOC_{ij}^{ss})\times VXE_{j}^{sr}\;+\sum_{i}\sum_{t\neq s}\hat{VSH_{t}^{s}}\times L_{ij}^{ts}\times VXE_{j}^{sr\;\;\;\;\;\;\;\;\;\;\;\;\;\;\;\;\;\;\;\;\;\;}$$

Within the DVA component, we compute the sectoral origin of value added, assuming that the sectoral origin of value added coincides with the exporting countries. Accordingly, the aggregated DVA component can be split by distinguishing between the values originating in (a) the domestic exporting sector (***direct exports***) and (b) other domestic sectors providing intermediate inputs to the domestic exporting sector (***indirect exports***). Indirect exports refers to the inputs from a particular sector traveling through the local production chain, i.e., not crossing any border before reaching the exporting sector.

(14) $$\sum_{j}DVA_{j}^{sr}=\sum_{i}\hat{VSH_{l}^{s}}\times LOC_{ii}^{ss}\times VXE_{i}^{sr}+\sum_{i}\sum_{j\neq i}\hat{VSH_{l}^{s}}\times LOC_{ij}^{ss}\times VXE_{i}^{sr}$$

It is worth noting that our approach is fundamentally consistent with that of Koopman et al. [18], applying the decomposition method of value-added trade to the multi-regional input–output table. However, we combine this value-added decomposition approach with a global CGE model to analyze the effects of trade policies, like the RCEP, on value-added trade.

### 3.3. The Reduction in Tariff and Non-Tariff Barriers

With the expansion of the GTAP model with the value-added decomposition, two policy scenarios with respect to the RCEP were established. The scenarios simulated the reduction in import tariffs and non-tariff barriers among the RCEP members. To calculate the actual reduction in import tariffs by 2030, a benefit of the RCEP, we compiled the relevant provisions and schedules of tariff reduction from the text of the RCEP agreement (the documents are available at http://fta.mofcom.gov.cn/rcep/rcep_new.shtml, 30 January 2024), which provides the benchmark and target tariff rates for the bilateral commodity trade among the members at the HS 10-digit code. We could not obtain the data on bilateral trade using the HS 10-digit code, so the average tariff rates were calculated using the HS 6-digit code. Then, we established the concordance between the seven aggregated sectors (see Appendix A) and the HS 6-digit code. Using the bilateral trade values from the UN COMTRADE [29] database as weights, we calculated the benchmark and target tariff rates for the bilateral trade of aggregated sectors among member countries. Finally, the reductions in the import tariff rates were obtained.

Next, the non-tariff barriers were gauged by the tariff equivalence, which was estimated based on the method suggested by Hummels et al. [35], i.e., by multiplying the time delays for crossing international borders from the Doing Business report [3] with ad valorem tariff equivalents of shipping delays estimated by Minor and Hummels [36]. It is worth noting that other studies also calculated the non-tariff barriers, e.g., those of Kee et al. [37] and Kee and Tang [38]; however, their indicators for non-tariff barriers could not be directly transferred to ad valorem tariff equivalents as the exogenous shock to the GTAP model.

The reductions in non-tariff barriers were much larger than the import tariffs imposed by RCEP members and India (Table 2). While the bilateral or multilateral FTAs have largely reduced the import tariffs across the RCEP members in the past, a major benefit of a series of FTAs, the non-tariff barriers are still a key challenge to prompting regional integration in Asia–Pacific economies. Hence, the reductions in import tariffs and non-tariff barriers in the RCEP would effectively stimulate commodity trade and affect value-added trade in Asia–Pacific economies, which has mostly been ignored in previous studies. It is worth mentioning that, due to the unavailability of data, this study did not consider the trade liberalization of service sectors from the RCEP agreement when calculating the shocks.

## 4. The Simulation Results for the Effects of the RCEP

### 4.1. Changes in Real GDP and Gross Trade

By cutting down tariff and non-tariff barriers, the RCEP would significantly expand the economies of Asia–Pacific countries involved in the agreement. Among them, ASEAN countries would jointly have the largest percentage increase in real GDP (1.96%, Table 3), which could be attributed to their relatively high non-tariff barriers before the RCEP. China’s real GDP would increase by 0.67%, followed by Korea (0.47%), New Zealand (0.46%), Australia (0.44%), and Japan (0.28%). In terms of absolute change in real GDP, China is projected to have the largest increase in real GDP by USD 69.21 billion, followed by ASEAN countries (USD 49.46 billion), Japan (USD 13.08 billion), Korea (USD 6.61 billion), Australia (USD 6.39 billion), and New Zealand (USD 0.91 billion). The absolute changes in GDP will depend on the percentage changes in real GDP and their economic size. However, countries that do not join the RCEP would experience losses in real GDP. The losses in real GDP of the European Union and the United States would reach USD 6.02 billion and USD 1.70 billion, respectively. India, which did not join the agreement in 2019, is projected to have the largest percentage decline in real GDP (0.06%).

Besides raising real GDP, the RCEP would also have significantly positive effects on the gross exports and imports of the member Asia–Pacific economies. The gross exports of China and ASEAN countries would increase significantly by over 3%, and those of Korea, Australia, and New Zealand would increase by over 2% (Table 3). In terms of absolute changes, China would have the greatest gross export increase of USD 105.56 billion due to its having the largest scale of exports in the world, followed by ASEAN countries (USD 49.12 billion), Korea (USD 18.12 billion), Japan (USD 18.07 billion), and Australia (USD 7.38 billion). While the percentage increase in gross exports for New Zealand is significant, the absolute increase is relatively small (USD 1.27 billion) because of the relatively small scale. On the other hand, because of the RCEP, New Zealand would have a significant increase in gross imports by 7.08%, followed by Japan (7.01%), ASEAN countries (6.80%), China (6.77%), Korea (6.52%), and Australia (5.74%), due to the reduced tariff and non-tariff barriers and the boosted economy (Table 3). In terms of absolute change, China would have the greatest increase in gross imports by USD 140.85 billion, as it has the second-largest worldwide imports, next only to the United States. The gross imports of ASEAN countries, Japan, Korea, and Australia would rise by USD 95.05 billion, USD 65.36 billion, USD 40.18 billion, and USD 15.42 billion, respectively.

Compared with the economies participating in the RCEP, gross imports of all the non-member economies would moderately decline, and the gross exports of some economies would increase. Among these countries, Taiwan would have the largest decrease in gross imports in terms of percentage change (3.34%), owing to economic degradation, followed by the United States (1.56%), India (1.09%), and Hong Kong (0.90%). The absolute damage to gross import would reach a significant level of USD 40.98 billion for the United States, followed by the European Union (USD 38.43 billion), the rest of the world (USD 37.27 billion), and Taiwan (USD 9.54 billion). In contrast, only Taiwan (−0.79%) and Hong Kong (−0.17%) would suffer from decreases in gross exports (Table 3), due to the loss of comparative advantage in the global market, which the possible benefits from the incremental demand for imported goods of RCEP members could not cover. In terms of absolute change, their decreases in gross exports will reach USD 2.82 billion and USD 0.32 billion. The gross exports of the United States, India, and the European Union would rise by 0.70%, 0.23%, and 0.14%, respectively, equivalent to increases in values of gross exports by USD 13.99 billion, USD 1.00 billion, and USD 9.96 billion. The gross exports from these countries would benefit from the incremental demand for imported goods of RCEP members.

**Figure A1:**
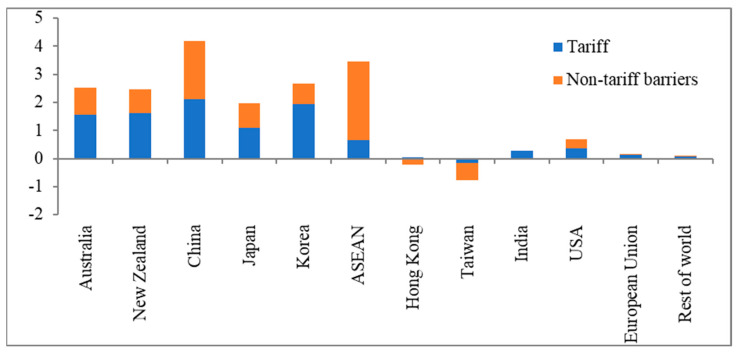
The effects of the RCEP on gross export by the reduction in tariff and non−tariff barriers.

We also found that reducing tariff and non-tariff barriers would have largely different impacts on the gross exports of RCEP members (see Appendix C, Figure A1). For Australia, New Zealand, Japan, and Korea, the increases in gross exports would mainly be from the reduction in tariffs. Reducing tariff and non-tariff barriers would have similar effects on China’s gross exports. For ASEAN countries, the increases in gross exports would mainly be driven by the reduction in non-tariff barriers. In addition, we compared the simulation results with previous studies [7,8,9,10,11]. The simulation results of this study are fundamentally consistent with these studies in terms of macroeconomic impacts (including GDP, total exports, total imports, and EV), verifying the rationale of the simulation.

### 4.2. The Origin Decomposition of the Changes in Value-Added Exports

The value-added exports embodied in gross trade are decomposed into three components: domestic value added (***DVA***), the double-counted term related to the domestic content (***DDC***), and the foreign content (***FVA***), as shown in Table 4. Notably, the sums of these components for the countries equal their gross exports in Table 3. Overall, the signs of changes in the components of value-added exports are consistent with those of gross exports. DVA exports are much smaller than gross exports. Unlike FVA exports, domestic value added is the revenue earned from trade and directly allocated to domestic labor, capital owners, and government, finally becoming a country’s wealth.

The effects of the RCEP on DVA exports are much different from those of gross exports in Asia–Pacific countries, which suggests that they are unable to obtain all the benefits embodied in their export flows. DVA exports would increase by USD 6.71 and 2.03 billion for Australia and New Zealand, respectively (Table 4), accounting for over 75% of the changes in their gross exports, indicating that they will benefit most from their export flows. For Australia and New Zealand, over 50% of exported goods are agricultural products, extraction products, and processed food, of which the value added is mostly produced domestically; the shares of FVA exports are relatively small. Meanwhile, the DVA exports would increase by USD 15.40 billion and USD 25.93 billion in Korea and ASEAN countries, respectively, accounting for less than 60% of the changes in gross exports. Over 40% of the benefits embodied in their export flows are attributed to their trade partners, resulting from their export-oriented economies, in which they import intermediates and export processed products. Interestingly, although China and Japan have large bodies of intermediate imports and processed product exports, their increases in DVA exports would reach USD 69.18 billion and USD 27.98 billion, respectively, accounting for nearly 70% of the changes in gross exports. Compared with Korea and ASEAN countries, China’s and Japan’s production of export products more highly depends on domestic-produced intermediates, owing to their relatively integrated industrial systems.

As for the economies that have not joined the RCEP, the decrease in DVA exports suggests negative effects on these countries’ real benefits, although several economies would have increases in gross exports. DVA exports would decline by USD 0.79 billion and USD 5.17 billion in Hong Kong and Taiwan, respectively, accounting for nearly 50% of the decreases in gross exports (Table 4). This reduction not only depresses the intermediate demand at home and abroad but also cuts down both DVA and FVA exports. In the meantime, the European Union and the rest of the world would have much larger decreases in DVA exports than FVAs, which may be attributed to the fact that their export products depend more heavily on domestically produced intermediates than on imports. It was surprisingly found that the FVA exports by the United States would decrease by USD 2.43 billion, which is larger than the decrease in DVA (USD 1.38 billion). Similarly, India’s FVA export decrease would be larger than the DVA reduction. These countries’ export production requires many imported intermediates with value addition originating abroad. Therefore, the losses in comparative advantage in the global market would cause relatively slight damages to their DVA exports.

### 4.3. The Sectoral Value-Added Exports and Imports

While the RCEP would have relatively small impacts on the export of agriculture, extraction products, and light manufacturing, it would significantly raise exports of processed food, textiles and clothing, and heavy manufacturing but hinder the export of services. As the import taxes and non-tariff barriers are relatively low for agriculture, extraction products, and light manufacturing for most RCEP members, the increases in these product exports would be relatively small. The exceptions are increased agricultural product exports from ASEAN countries and light manufacturing from China due to the existing non-tariff barriers. Compared with these products, the import taxes and non-tariff barriers for processed food, textiles and clothing, and heavy manufacturing are so significant that RCEP would improve their exports significantly, making them the major sources of increased value-added exports. For example, the exports of heavy manufacturing are projected to increase by USD 79.88 billion, USD 34.77 billion, USD 27.62 billion, and USD 53.89 billion for China, Japan, Korea, and ASEAN countries, respectively (Table 5). In contrast, the export of services from RCEP members would decline by USD 0.39–23.95 billion, except for Australia. The economic expansion caused by the agreement would increase the demand for services in RCEP member countries and consequently raise the prices of services, largely overwhelming the reductions in import taxes and non-tariff barriers. As a result, the RCEP would raise commodity exports but hinder the export of services by the member countries.

Except for extraction products, the imports of tradable products from RCEP members would rise. The largest increase would occur in processed foods, heavy manufacturing, and services, resulting from reduced import taxes and non-tariff barriers among RCEP countries (Table 5). China’s exports of heavy manufacturing would increase by USD 111.46 billion, followed by Japan (USD 40.55 billion), Korea (USD 27.28 billion), ASEAN countries (USD 13.87 billion), and Australia (USD 10.79 billion). However, the import of extraction products would decline, except for ASEAN countries.

From the perspective of value-added decomposition, the DVA exports in agricultural products and processed food were estimated, revealing the benefits gained from the RCEP (Table 6). DVA exports of agricultural products and processed food from most RCEP members would increase significantly. For example, as the biggest winner, the DVA exports of agricultural products and processed food from China would rise by USD 1350.5 and USD 4728.3 million, respectively. Even for Japan, its DVA exports in agricultural products and processed food would increase by USD 75.7 and 94.1 million, respectively. These results show that the closely integrated global value chain boosts the food economy for RCEP members. The exceptions are agricultural products from New Zealand (USD −22.4 million) and processed food from ASEAN countries (USD −3960.2 million). For New Zealand, the negative impact on agricultural DVA exports would be covered by the significant increase in DVA exports of processed food. Similarly, the increased agricultural DVA exports from ASEAN countries would exceed the reduction in DVA exports in processed food. In addition, the FVA exports of RCEP members would also increase, indicating that non-RCEP members would also gain some economic benefits from the increasingly integrated food value chain in Asia–Pacific areas.

## 5. Discussion and Conclusions

This paper uses a CGE modeling framework (the GTAP model) for analysis, with an extension module for the origin decomposition of value-added flows embodied in gross trade. Compared to previous studies, on the one hand, the effects of the RCEP are examined from a value-added trade perspective, separating the DVA exports that represent the real benefits gained by Asia–Pacific economies. On the other hand, this paper examines the effects of the RCEP on food and non-food products, adding a novel viewpoint to the literature.

Our results suggest that by cutting down tariff and non-tariff barriers, the RCEP would significantly stimulate the GDP growth of Asia–Pacific countries and have significantly positive effects on gross exports and imports. Compared with the economies in the RCEP, gross exports and imports of the non-member economies would moderately decline. The potential benefit of the RCEP will be overestimated if we ignore the origin of value added and measure the benefits by gross exports. Due to the reduction in tariff and non-tariff barriers, the domestic components of bilateral value-added trade would also increase, which indicates the increasingly integrated value chain between RCEP members. Due to the trade diversion effect, value-added exports of RCEP members to non-RCEP economies would consistently decline. Import taxes and non-tariff barriers of processed food, textiles and clothing, and heavy manufacturing are more significant because the RCEP economies would significantly improve their value-added exports. Except for extraction products, imports of tradable products from RCEP members would also rise. The domestic component of value-added exports in agricultural products and processed food from most RCEP members would increase significantly, indicating that the food economy in RCEP members would be boosted by the closely integrated global value chain.

Three caveats are also worth mentioning in the analysis presented here. First, this study excludes the liberalization of services and foreign direct investment, which cautions that our results are conservative. The impact of the liberalization of foreign direct investment that comes from participation in an FTA is not accounted for, nor is the potential of increased productivity effects associated with greater competition incentives to innovate from enhanced competitive pressure [39]. Thus, welfare gains are, more often than not, underestimated. In addition, the GTAP model does not incorporate the financial sector. Hence, the effects are limited to the real economy, which requires the finance module extension to the GTAP model. Second, the possibility of India’s future participation should be given more attention in future studies. India is apprehensive about greater market access to RCEP members, such as China, Australia, and New Zealand, which may harm its key manufacturing sectors, such as steel and textiles. However, the potential benefits to the current 15 member countries may attract India to restart talks on the deal. Moreover, the political institutions, cultural values, and underlying tensions among stakeholders in India should be considered in future studies [40]. Third, a series of previous studies, such as those of Chen et al. [41], Yang et al. [42], and Duan et al. [43], proved that without distinguishing processing exports, the domestic value added embodied in exports will be overestimated, especially for China. However, as this study uses the GTAP database, it cannot consider processing exports, which should be paid more attention in future studies.

## Figures and Tables

**Table 1:** Structure of commodity trade among RCEP countries and India in 2018 (USD billion).

	China	Japan	Korea	ASEAN	Australia	New Zealand	India
	Exports						
Agriculture	21.5	1.2	1.1	17.2	15.7	6.4	10.9
Extraction	7.5	0.4	0.5	80.7	113.3	0.7	5.1
Processed food	55.9	6.2	7.0	98.8	19.5	20.9	26.6
Textiles and clothes	282.0	7.7	13.4	31.6	0.7	0.4	36.0
Light manufacturing	174.8	7.4	6.4	42.7	3.1	2.5	8.9
Heavy manufacturing	1945.6	669.5	576.3	883.3	55.0	6.2	234.3
	Imports						
Agriculture	82.0	21.2	10.4	27.5	2.0	0.8	10.5
Extraction	446.3	172.6	135.9	102.3	11.9	3.1	214.8
Processed food	61.9	53.7	22.8	55.3	13.9	4.3	16.3
Textiles and clothes	26.4	39.5	16.7	24.8	10.3	2.0	7.6
Light manufacturing	61.7	33.0	16.4	29.0	12.5	2.4	12.2
Heavy manufacturing	1280.5	416.5	332.9	915.5	177.0	30.5	353.6

Source: UN COMTRADE, 2020 [29].

**Table 2:** The average reductions in the rates of import tariff and non-tariff barriers for RCEP members (percentage points).

	Import Tariffs	Non-Tariff Barriers		
		Exports	Imports	
Australia	−1.30	−0.53	−1.38	
New Zealand	−1.02	−0.65	−0.84	
China	−1.21	−1.40	−3.21	
Japan	−0.57	−0.92	−0.80	
Korea	−1.21	−0.47	−0.13	
ASEAN	−0.13	−1.61	−3.05	

Note: The import tariffs and non-tariff barriers are averaged and weighted by bilateral commodity trade values. Source: Authors’ calculations.

**Table 3:** The effects of the RCEP on national GDP and gross trade.

	GDP		Gross Exports		Gross Imports	
	% Change	Absolute Change *	% Change	Absolute Change	% Change	Absolute Change
Australia	0.44	6.39	2.53	7.38	5.74	15.42
New Zealand	0.46	0.91	2.48	1.27	7.08	3.63
China	0.67	69.21	4.18	105.56	6.77	140.85
Japan	0.28	13.08	1.96	18.07	7.01	65.36
Korea	0.47	6.61	2.67	18.12	6.52	40.18
ASEAN	1.96	49.46	3.47	49.12	6.80	95.05
Hong Kong	0.00	0.00	−0.17	−0.32	−0.90	−1.66
Taiwan	−0.03	−0.16	−0.79	−2.82	−3.34	−9.54
India	−0.06	−1.27	0.23	1.00	−1.09	−5.65
USA	−0.01	−1.70	0.70	13.99	−1.56	−40.98
European Union	−0.03	−6.02	0.14	9.96	−0.54	−38.43
Rest of world	−0.03	−5.49	0.10	5.34	−0.70	−37.27

* The absolute changes in GDP are in USD billion. Source: Authors’ simulations.

**Table 4:** The effects of the RCEP on value-added exports (USD billion).

	DVA	DDC	FVA
Australia	6.71	0.03	2.45
New Zealand	2.03	0.00	0.64
China	67.18	2.94	31.25
Japan	27.98	0.38	11.64
Korea	15.40	0.26	14.80
ASEAN	25.93	0.62	14.08
Hong Kong	−0.79	0.00	−0.64
Taiwan	−5.17	−0.05	−4.08
India	−0.96	−0.01	−1.18
USA	−1.38	−0.07	−2.43
European Union	−21.36	−0.57	−10.92
Rest of world	−24.01	−0.78	−5.78

Source: GTAP simulation.

**Table 5:** The changes in value-added exports and imports by sector (USD billion).

	Australia	New Zealand	China	Japan	Korea	ASEAN	Hong Kong	Taiwan	India	USA	European Union	Rest of World
Exports												
Agriculture	0.25	−0.02	1.49	0.09	0.17	10.52	0.00	0.01	0.05	−0.97	−0.46	−1.92
Extraction	−1.46	−0.02	1.56	0.15	0.07	−2.67	0.01	0.00	−0.09	−0.48	0.01	−5.77
Processed food	1.54	2.14	5.56	0.12	0.54	−4.97	−0.02	0.08	0.21	0.77	0.17	0.47
Textiles and clothes	0.06	−0.02	9.36	4.62	3.26	5.90	−0.25	−1.01	−0.99	−0.63	−2.68	−2.50
Light manufacturing	0.13	0.00	5.34	1.08	0.73	1.90	−0.06	−0.04	−0.07	−0.49	−1.57	−1.70
Heavy manufacturing	7.16	0.98	79.88	34.77	27.62	53.89	−1.94	−10.06	−3.14	−12.55	−43.45	−27.56
Services	1.51	−0.39	−1.82	−0.84	−1.92	−23.95	0.82	1.72	1.88	10.47	15.14	8.40
Imports												
Agriculture	0.07	0.07	7.09	0.86	0.36	3.63	−0.01	−0.04	−0.21	−0.54	−0.90	−1.16
Extraction	−0.11	−0.08	−2.16	−2.38	−1.01	4.87	−0.12	−0.17	−1.17	−1.55	−3.92	−0.88
Processed food	0.80	0.40	5.04	4.13	1.36	4.01	−0.05	−0.20	−0.27	−1.83	−2.49	−4.26
Textiles and clothes	1.36	0.26	8.22	5.85	3.10	3.00	−0.01	−0.16	−0.12	−1.91	−2.22	−2.24
Light manufacturing	0.99	0.19	3.81	3.95	1.10	1.25	−0.03	−0.15	−0.12	−1.68	−2.32	−1.75
Heavy manufacturing	10.79	2.22	111.46	40.55	27.78	13.87	−0.84	−6.29	−3.34	−29.01	−29.23	−32.35
Services	1.47	0.58	10.61	9.26	6.04	18.13	−0.30	−1.34	−0.71	−6.53	−16.30	−9.92

Source: GTAP simulation.

**Table 6:** The changes in agriculture and food value-added exports (USD million).

	Agriculture			Processed Food		
	DVA	DCC	FVA	DVA	DCC	FVA
Australia	189.8	1.6	57.4	1321.9	2.6	218.5
New Zealand	−22.4	0.0	4.2	1760.7	0.6	380.7
China	1350.5	8.6	130.7	4728.3	33.8	802.6
Japan	75.7	0.4	14.9	94.1	0.6	27.2
Korea	134.8	0.3	32.4	385.5	1.2	157.8
ASEAN	9513.5	18.1	992.0	−3960.2	−5.3	−1001.2
Hong Kong	−1.0	0.0	−0.4	−14.7	0.0	−4.5
Taiwan	8.0	0.0	1.2	46.2	0.0	32.0
India	45.1	0.0	2.4	190.6	0.0	22.9
USA	−871.5	−2.6	−94.5	701.5	1.9	65.2
European Union	−399.0	−2.8	−53.8	210.0	−4.5	−32.9
Rest of world	−1768.4	−16.6	−139.8	445.0	−0.9	25.3

Source: GTAP simulation.

**Figure A2:**
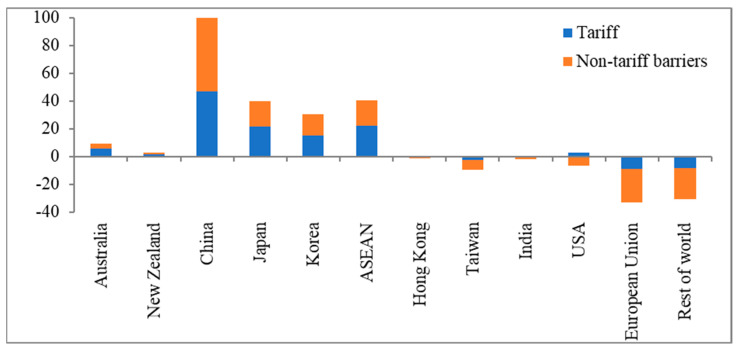
The effects of the RCEP on value-added exports by the reduction in tariff and non−tariff barriers (USD billion).

## Data Availability

The original contributions presented in the study are included in the article; further inquiries can be directed to the corresponding author.

## Appendix A. The Sectoral and Regional Concordance of the GTAP Model

No.	Aggregated regions	Original GTAP regions
1	Australia	aus
2	New Zealand	nzl
3	China	chn
4	Hong Kong	hkg
5	Japan	jpn
6	Korea	kor
7	Taiwan	twn
8	ASEAN countries	brn, khm, idn, lao, mys, phl, sgp, tha, vnm, xse
9	India	ind
10	USA	usa
11	European Union	aut, bel, bgr, hrv, cyp, cze, dnk, est, fin, fra, deu, grc, hun, irl, ita, lva, ltu, lux, mlt, nld, pol, prt, rou, svk, svn, esp, swe, gbr
12	ROW	xoc, mng, xea, bgd, npl, pak, lka, xsa, can, mex, xna, arg, bol, bra, chl, col, ecu, pry, per, ury, ven, xsm, cri, gtm, hnd, nic, pan, slv, xca, dom, jam, pri, tto, xcb, che, nor, xef, alb, blr, rus, ukr, xee, xer, kaz, kgz, tjk, xsu, arm, aze, geo, bhr, irn, isr, jor, kwt, omn, qat, sau, tur, are, xws, egy, mar, tun, xnf, ben, bfa, cmr, civ, gha, gin, nga, sen, tgo, xwf, xcf, xac, eth, ken, mdg, mwi, mus, moz, rwa, tza, uga, zmb, zwe, xec, bwa, nam, zaf, xsc, xtw
No.	Aggregated sectors	Original GTAP sectors
1	Agriculture	pdr, wht, gro, v_f, osd, c_b, pfb, ocr, ctl, oap, rmk, wol, frs, fsh
2	Extraction	coa, oil, gas, oxt
3	Processed food	cmt, omt, vol, mil, pcr, sgr, ofd, b_t
4	Textiles and clothes	tex, wap
5	Light manufacturing	lea, lum, ppp
6	Heavy manufacturing	p_c, chm, bph, rpp, nmm, i_s, nfm, fmp, ele, eeq, ome, mvh, otn, omf
7	Services	ely, gdt, wtr, cns, trd, afs, otp, wtp, atp, whs, cmn, ofi, ins, rsa, obs, ros, osg, edu, hht, dwe

## Appendix B. The Key Parameters of the GTAP Model

**Table A1:** The value of substitution elasticity for domestic and imported commodities (ESUBD) and substation elasticity for regional allocation of imports (ESUBM).

	ESUBD	ESUBM
Agriculture	2.35	4.80
Extraction	5.70	13.01
Processed food	2.48	4.97
Textiles and clothes	3.73	7.44
Light manufacturing	3.27	6.93
Heavy manufacturing	3.45	7.22
